# Supplementary figures and images for: A Dual Regulatory Mechanism of Hormone Signaling and Fungal Community Structure Underpin Dendrobine Accumulation in Dendrobium nobile
Source: Biomolecules. 2025 Sep 26;15(10):1366. doi: 10.3390/biom15101366 (PMC12562096; doi:10.3390/biom15101366)

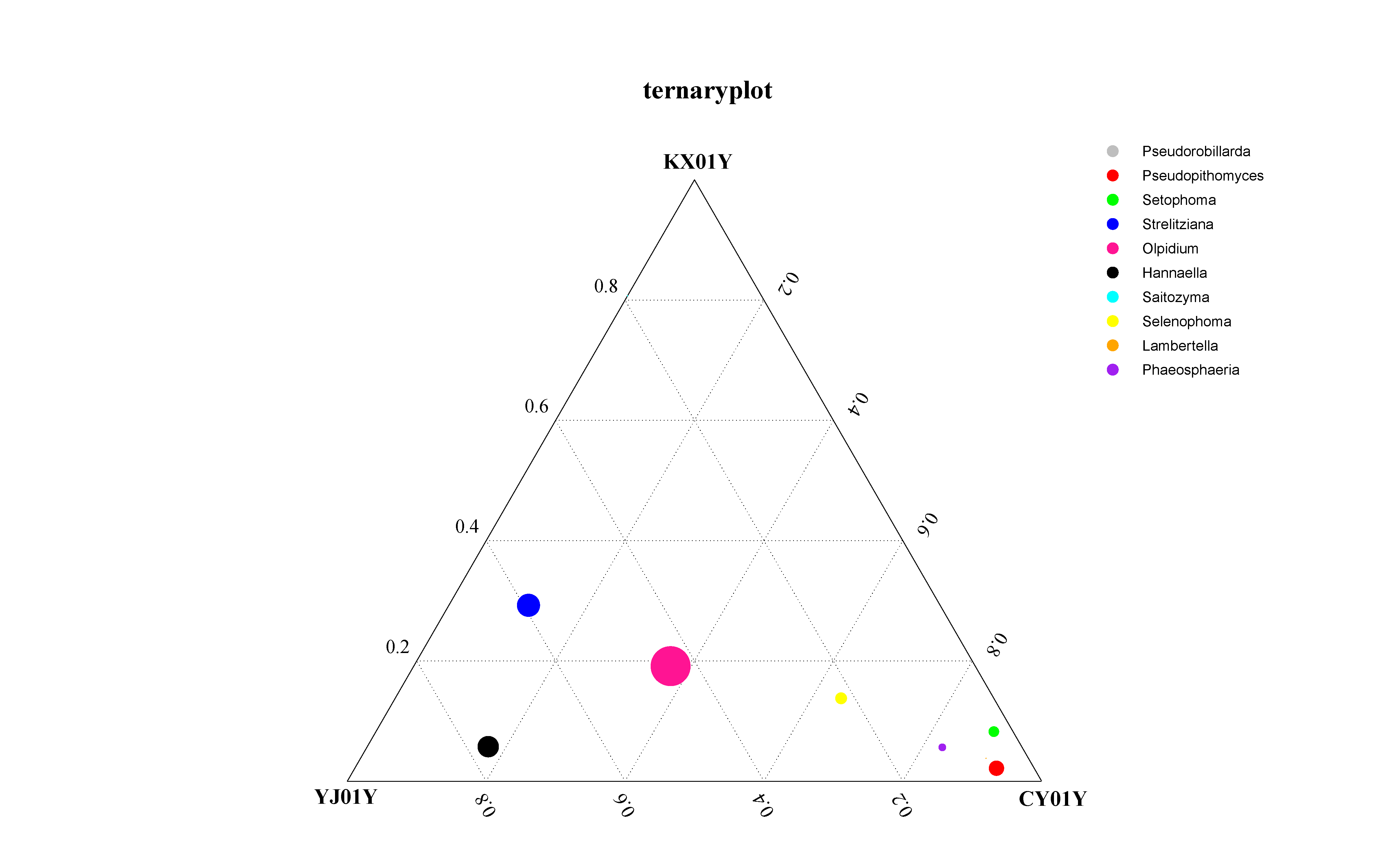

Supplement: Supplementary file 1 [file biomolecules-15-01366-s001.zip › Supplementary Figure S1. Distribution of Endophytic Fungi in Stems of Dendrobium nobile from Different Semi-Wild Cultivation Bases - Ternary Phase Diagram (Genus Level)​.tif]

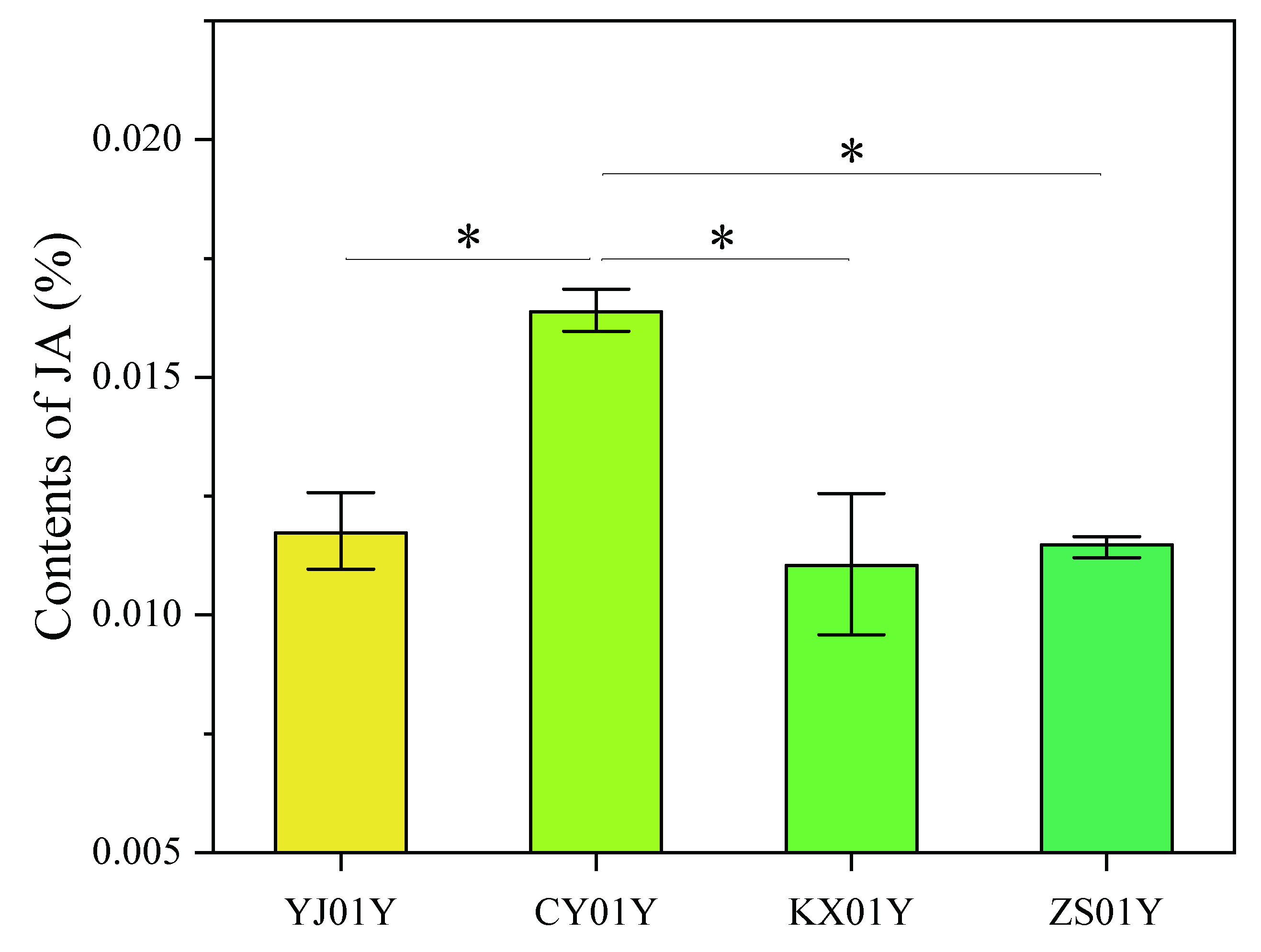

Supplement: Supplementary file 1 [file biomolecules-15-01366-s001.zip › Supplementary Figure S2. Content of the Plant Hormone Jasmonic Acid in Stems of Dendrobium nobile from Different Semi-Wild Cultivation Bases.tif]
